# Loneliness within couples coping with cancer: prevalence, associations with physical and mental health, and potential benefits of dyadic exercise

**DOI:** 10.3389/fragi.2026.1812899

**Published:** 2026-04-17

**Authors:** Kerri Winters-Stone, Sydnee A. Stoyles, Nathan Dieckmann, Alexandra O. Sokolova, Julie N. Graff, Arthur Hung, Shirlene Wang, Karen S. Lyons

**Affiliations:** 1 Division of Oncological Sciences, Portland, OR, United States; 2 Knight Cancer Institute, Oregon Health & Science University, Portland, OR, United States; 3 School of Nursing, Oregon Health & Science University, Portland, OR, United States; 4 School of Nursing, Boston College, Boston, MA, United States

**Keywords:** caregiver, dyad, exercise, physical activity, social support

## Abstract

**Introduction:**

Cancer survivors and intimate care partners coping with cancer may each experience loneliness that impacts their health, but research within couples and potential benefit of interventions is scarce. METHODS: We added the Cancer Loneliness (CL) scale to measures of anxiety, depressive symptoms, pain and social and physical function mid-way through the Exercising Together trial (NCT03630354) in couples coping with breast (BC; n = 44) or prostate cancer (PC; n = 75). The prevalence of CL scores >1 indicated at least some loneliness and we ran correlations between CL and measures of social, physical and mental health. Within PC couples we explored the potential benefits of different types of dyadic exercise programs (supervised, group partnered exercise, supervised separate groups of survivors or partners, or unsupervised independent exercise) on CL over 6 months.

**Results:**

96% of breast cancer survivors (BCS) and 77% of prostate cancer survivors (PCS) reported CL at baseline. CL was positively associated with anxiety (rho = 0.44, 0.77), depressive symptoms (rho = 0.60, 0.67) and pain (rho = 0.34, 0.23) and inversely associated with social functioning (rho = −0.54, −0.55) among BCS and PCS, respectively (all p < 0.01). CL was inversely associated with physical functioning in BCS (rho = −0.35), but not PCS. Among care partners, 74%–77% of care partners for each BCS and PCS reported CL related to their partner’s cancer at baseline. CL was positively associated with anxiety (rho = 0.43, 0.30) and depressive symptoms (rho = 0.62, 0.45) among BC and PC care partners, respectively (all p < 0.01). CL was inversely associated with social functioning in BC partners only (rho = −0.45, p < 0.01), but not with physical functioning in either group. Within PC couples, there was a significant (inverse) interaction between change in CL over six-months of exercise and baseline loneliness. Declines in CL for both PCS and care partners were steepest in the exercise program where they trained with other PCS or care partners.

**Conclusion:**

Relationships may not offer enough social support to avoid feelings of loneliness associated with cancer that in turn affects the physical, emotional and social health of each partner. However, exercise may help rebuild connection within the couple and be an avenue for informal social support that together could reduce loneliness.

**Clinical Trial Registration:**

[clinicaltrials.gov]: identifier, [NCT03630354].

## Introduction

Loneliness is considered an innate evolutionary warning signal, similar to hunger or pain (Cacioppo et al., 2018) and is a worldwide public health problem (Office of the Surgeon General, 2026). It is a subjective feeling of being alone characterized by an imbalance between desired social contacts and actual social contacts. Recognized as an established risk factor for depression and anxiety (Beutel et al., 2017), loneliness also correlates with poor physical health in the general population (Shaw et al., 2021), carrying risks for premature death equivalent to those attributable to smoking, obesity and inactivity (Office of the Surgeon General, 2026). Loneliness can be experienced socially, emotionally and existentially and people with cancer often report heightened feelings of loneliness. The cancer experience contribute to patients feeling alone and misunderstood (Hawkley et al., 2010), and in people with cancer, loneliness is linked to poor mental and physical health (Hawkley and Cacioppo, 2003; Cacioppo et al., 2010; Cacioppo and Hawkley, 2009; Hawkley et al., 2010; Jaremka et al., 2014; Jaremka et al., 2013). Since cancer is typically diagnosed at an older age (Bluethmann et al., 2016) and the risk of loneliness increases with age due to shrinking social networks (Huxhold and Fiori, 2024/06), the combined effects of aging and cancer amplify risk.

Marriage is considered to be a protective factor against loneliness (Beutel et al., 2017; National Academies of Sciences and Medicine, 2020), but that effect may be dampened within couples who are coping with a chronic illness such as cancer. The toll of an illness may erode the support and emotional intimacy within romantic relationships that protect a couple from threats to their mental and physical wellbeing (Niedling and Hamel, 2023). Within the couple, the partner experiencing an acute or chronic illness may avoid discussing their own emotional needs in order to protect their partner, maintain normal household routines, and keep up an appearance of normality for loved ones (Emslie et al., 2009). People with cancer who do not have other trusted persons to confide in about their worries and fears, who choose not to confide in a partner, or who receive negative support from friends and family are at risk for poor psychosocial and overall wellbeing (Eymech et al., 2022; Helgason et al., 2001). Intimate care partners of people with cancer also report feelings of loneliness that may be related to distress and the time demands of caregiving coupled with the same desire to protect their ill partner from their own emotional burden. Caregiving demands can isolate care partners from formal (i.e., support group) and informal (i.e., friends) social networks, which regardless may provide insufficient or mismatched support, and because support is often disproportionately directed to the ill partner (Ross et al., 2020). Anticipatory grief over the potential loss of a partner and grief over the life planned together prior to cancer may further isolate partners from one another. Loneliness can have a similar negative impact on care partner physical and mental health as it does on their ill partner, particularly for older caregivers who may have their own health challenges (Ross et al., 2020; Liu et al., 2024). High levels of psychological distress can have long-term negative consequences for both partners and compromise the ability of the spouse or care partner to provide quality care (Northouse et al., 2001; Rohrbaugh et al., 2006).

Existing recommendations to reduce loneliness in individuals include cognitive and psychological reframing, individual therapy, befriending, and resilience training. Social support is widely acknowledged as a protective factor in ameliorating the psychological burden associated with cancer (Oliffe et al., 2009); however, there is a paucity of controlled trials aimed to reduce loneliness in people with cancer and these mostly consist of an education or psychosocial intervention (Wilson et al., 2024). Results are mixed with some interventions reducing loneliness and others having no effect. Notably, there are no published controlled trials characterizing loneliness specific to caring for someone with cancer nor of reducing loneliness in intimate care partners of cancer survivors. In people without cancer, exercise-based strategies that integrate activities to enhance social engagement among participants have been effective at reducing social isolation (Yu et al., 2023). Large longitudinal cohort studies have also reported that physical activity can moderate the harmful impact of chronic illness on loneliness (Infurna et al., 2026). If delivered to the couple, exercise could simultaneously alleviate social isolation and loneliness in both survivors and care partners by promoting a shared experience and connection within the dyad, while increasing physical health and emotional wellbeing. Likewise, delivering exercise in a group format with others sharing a similar life challenge could provide a source of informal social support that people may not find elsewhere. Through a secondary analysis of our Exercising Together clinical exercise trial we addressed the following aims: 1) to describe cancer loneliness among cancer survivors and their intimate care partners coping with breast or prostate cancer; 2) examine the relationship between cancer loneliness and mental and physical health in cancer survivors and their intimate care partners and 3) explore the potential benefits of exercise on loneliness within couples who participated in structured exercise programs that varied in the level of social support from peers and the nature of dyadic interactions.

## Methods

**FIGURE 1:**
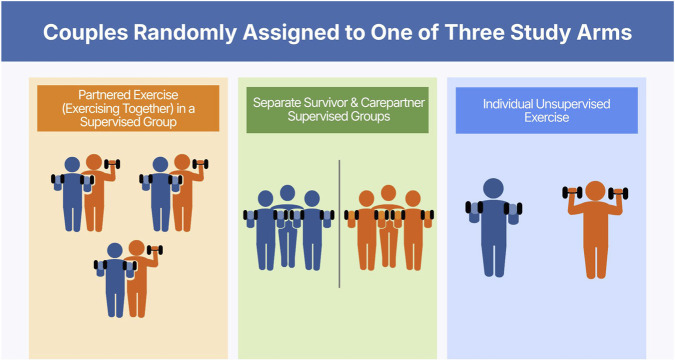
Depiction of study arms from the parent trial.

The Exercising Together trial (NCT03630354) was a randomized controlled trial comparing three dyadic exercise programs on relationship, physical and mental health in couples (survivor and intimate spouse/partner) coping with breast (BC) or prostate (PC) cancer where the survivor completed definitive treatment within the past 3 years and each partner was between 40–85 years old (Winters-Stone et al., 2021). Full trial eligibility is described elsewhere (Winters-Stone et al., 2021). Participants were primarily recruited through the Oregon State Cancer Registry, but upon shifting to live, remote intervention delivery and assessment couples could be recruited throughout the U.S. The primary goal of the parent intervention was to determine the unique benefits of having couples exercising as a team in a program called Exercising Together^©^ compared to the benefits they receive from exercising in a group with other people and from engaging in a shared exercise behavior. To answer this question couples were randomized to one of three dyadic exercise programs (Figure 1): 1) Supervised group exercise where couples engaged in a partnered exercise program (Exercising Together^©^) with other couples (up to 10 couples per group), 2) Supervised group exercise where survivors and care partners participated in separate classes with other survivors or care partners (up to 10 participants per group), respectively, or 3) Unsupervised independent exercise where both partners were taught an individual exercise program that they engaged in on their own at home. All groups were expected to exercise twice a week for 6 months. While supervised group exercise classes were initially held in person, about one-quarter of the way through the trial the COVID-19 pandemic hit and for the remainder of the trial classes were held on the virtual platform Zoom. In live remote classes, participants engaged in 15 min of informal social time before each exercise session to replace the group engagement that occurred organically in person.

We added a questionnaire about cancer loneliness midway through the trial to planned assessments collected at baseline, mid-intervention (3 months), post-intervention (6 months), and 6 month follow up (12 months). A cancer specific loneliness measure captures the unique impact of cancer on a person’s desired and actual social connections, that is different from feelings of general loneliness (Adams et al., 2017). For example, people with cancer may experience negative reactions when trying to discuss their cancer with others and/or they may experience existential concerns that others may not understand. Additional assessments already part of the parent study included self-report demographics (including height and weight) and clinical history, and patient-reported outcomes of anxiety (PROMIS) with scores ranging from 0–100 where higher scores indicate higher anxiety (Cella et al., 2019); depressive symptoms (CES-D) with scores ranging from 0–60 with higher scores indicating higher depressive symptomology (Radloff, 1977); and social and physical function and bodily pain (SF-36 subscales) with scores ranging from 0–100 where higher scores indicate better functioning and less bodily pain (Ware and Sherbourne, 1992). The 7-item Cancer Loneliness Scale (CLS) (Adams et al., 2017) measures cancer loneliness by asking how often people experience a feeling of loneliness specific to having cancer, such as “how often do you feel others cannot provide the support your need to deal with your cancer?” on a Likert scale of 1 (never) to 5 (always). Responses are averaged, where higher scores indicate greater feelings of cancer loneliness. We adapted the CLS for care partners by replacing the phrase “your cancer” to “your partner’s cancer”.

We conducted an ancillary analysis on the subsample of survivors and partners completing the CLS at baseline. Among PC couples, 75 prostate cancer survivors (PCS) and 73 PC partners completed the CLS at baseline and among BC couples, 44 breast cancer survivors (BCS) and 43 BC partners completed baseline CLS. Since the analysis was limited to individual-level outcomes, we included all cases with complete baseline data, even if one partner was excluded because they did not complete the CLS. We first calculated the prevalence of cancer loneliness as the percentage of survivors or care partners reporting CLS scores >1, indicating they experienced some level of cancer loneliness and determined the association of cancer loneliness with baseline demographics and clinical characteristics (Aim 1), and then with self-report mental and physical health using Spearman’s correlations (Aim 2). We used a piecewise mixed-effects model to estimate changes in cancer loneliness over the course of the six-month exercise programs combined and from the end of the program to 6-month follow-up as a function of baseline cancer loneliness (Aim 3) using all available observations from the subsample and stratified by survivors and partners (k = 270 and 261 observations, respectively). Data completion rates on the primary outcome of cancer loneliness at baseline, 3, 6 and 12 months were as follows: PCS: n = 75, 67, 63, 65; PC partners: n = 73, 65, 63, 66. Due to the small sample sizes within each study arm we did not conduct statistical analyses comparing the three exercise groups but made visual inferences from figures of group changes over time within survivors and care partners. The cancer loneliness survey was inadvertently missed at follow-up for BC couples; thus, longitudinal analyses are only available for PC couples.

## Results

### Study sample

PCS (n = 75) and care partners (n = 73) who completed the CLS at baseline were an average of 69.4 ± 6.5 and 67.0 ± 6.8 years old, respectively, mostly white (93% and 89%), well educated (83% and 70% with Bachelor’s degree or higher), and partnered for 36.0 ± 3.7 years, with 40% living in rural areas. PCS were an average of 20 ± 9.2 months past their cancer diagnosis with the majority having surgery (72%), 39% having radiation therapy and 28% currently on androgen deprivation therapy (ADT) (Table 1). PCS had a higher level of comorbidities compared to their care partners (Charleson comorbidity Index (CCI) = 2.8 ± 1.1) and 0.9 ± 1.8, respectively), but similar BMI (29.1 ± 4.5 and 28.1 ± 5.8). BC survivors (BCS; n = 44) and partners (n = 43) were an average of 59.3 ± 9.3 and 62.2 ± 10.5 years old, respectively, mostly white (89% and 86%), well educated (73% and 64% with bachelor’s degree or higher), and partnered for 21.1 ± 12.6 years, with 23% living in rural areas. BCS were an average of 21.6 ± 6.7 months past their cancer diagnosis with the majority having surgery (96%), 71% having radiation therapy, 48% having chemotherapy and 71% on hormone therapy. BCS had a higher level of comorbidities compared to their care partners (Charleson comorbidity Index (CCI) = 2.8 ± 0.9 and 1.1 ± 1.5, respectively), but similar BMI (29.7 ± 6.9 and 31.8 ± 7.9).

**TABLE 1:** Baseline demographic, health and clinical characteristics by cancer type and role (survivor or partner).

Characteristic	Prostate cancer couples				Breast cancer couples			
	Survivor		Partner		Survivor		Partner	
	(n = 75)		(n = 73)		(n = 44)		(n = 43)	
	Mean (SD) or %	Range	Mean (SD) or %	Range	Mean (SD) or %	Range	Mean (SD) or %	Range
*Demographics*								
Time in relationship (years)	36.0 (13.7)	6.0–56.0	36.0 (13.7)	6.0–56.0	21.1 (12.6)	1.9–54.0	21.1 (12.6)	1.9–54.0
Age	69.4 (6.5)	51.3–79.4	67.0 (6.8)	44.5–77.7	59.3 (9.3)	37.8–78.3	62.2 (10.5)	40.8–80.5
Female	0.0%	​	98.6%	​	100.0%	​	9.3%	​
Race								
White/Caucasian	93.3%	​	89.0%	​	88.6%	​	86.1%	​
Black or african american	0.0%	​	0.0%	​	4.6%	​	4.7%	​
Asian	4.0%	​	6.9%	​	2.3%	​	2.3%	​
Native Hawaiian/Pacific Islander	1.3%	​	0.0%	​	0.0%	​	0.0%	​
Native american/Alaskan native	0.0%	​	0.0%	​	0.0%	​	0.0%	​
Multiracial	1.3%	​	1.4%	​	2.3%	​	4.7%	​
Other	0.0%	​	0.0%	​	0.0%	​	2.3%	​
Hispanic	0%	​	1%	​	2%	​	7%	​
Bachelor’s degree or higher	82.7%	​	69.9%	​	72.7%	​	67.4%	​
Work outside the home	29.3%	​	27.4%	​	61.4%	​	58.1%	​
Income								
<$25k	2.7%	​	1.4%	​	2.3%	​	2.3%	​
$25–50k	5.3%	​	5.5%	​	11.4%	​	11.6%	​
$50–100k	38.7%	​	27.4%	​	27.3%	​	30.2%	​
$100–150k	24.0%	​	37.0%	​	20.5%	​	23.3%	​
>$150k	25.3%	​	24.7%	​	36.4%	​	32.6%	​
Rural/Frontier residence	40%	​	40%	​	23%	​	26%	​
*Health and Clinical Status*								
Comorbidities (charleson)	2.8 (1.1)	2.0–6.0	0.9 (1.8)	0.0–11.0	2.8 (0.9)	2.0–5.0	1.1 (1.5)	0.0–7.0
BMI (kg/m^2^)	29.1 (4.5)	17.8–41.5	28.0 (5.8)	18.4–55.0	29.7 (6.9)	20.9–54.8	31.8 (7.9)	19.3–59.2
Time since diagnosis (mos.)	20.0 (9.2)	5.0–37.0	​	​	21.6 (7.7)	4.0–36.0	​	​
Stage^c^								
0	2.7%	​	​	​	13.6%	​	​	​
I	5.3%	​	​	​	43.2%	​	​	​
II	36.0%	​	​	​	31.8%	​	​	​
III	41.3%	​	​	​	11.4%	​	​	​
Treatment history								
Surgery	72.0%	​	​	​	95.5%	​	​	​
Radiation	38.7%	​	​	​	70.5%	​	​	​
Chemotherapy	1.3%	​	​	​	47.7%	​	​	​
ADT^a^	28.0%	​	​	​	​	​	​	​
AI/SERM^b^	​	​	​	​	71.4%	​	​	​

^a^ ADT: androgen deprivation therapy.

^b^ AI/SERM: Aromatase Inhibitor/Selective Estrogen Receptor Modulator.

^c^ Numbers may not add up to 100% because of unknown stage, but no survivor had a history of Stage IV, disease.

### Cancer loneliness among PC and BC couples

At baseline, 77% of PCS and 96% of BCS reported some level of cancer loneliness (i.e., CLS score >1 on a 1-5 scale) with an average score of 1.7 ± 0.7 and 2.3 ± 0.8, respectively (Table 2). Among PCS cancer loneliness was inversely associated with age (rho = −0.30; p = 0.01) and positively associated with ADT use (CL = 2.0 ± 0.9 for ADT and 1.6 ± 0.6 for non-ADT; p = 0.04), but not with other treatments, time since diagnosis, comorbidities, race, ethnicity, education, employment, income, rural residence, nor time in their relationship (Tables 2, 3). There were positive associations between cancer loneliness and time since diagnosis among BCS where loneliness was higher for those further away from diagnosis (rho = 0.31; p = 0.04), but no associations with other baseline demographic or clinical variables. At baseline, 74% of PC and 77% of BC partners reported some level of loneliness related to their partner’s cancer with an average score of 1.8 ± 0.7 for both groups (Table 2). Among PC partners, cancer loneliness was inversely associated with education where loneliness was higher among those with less education (p = 0.02), but no other baseline demographic (Table 3). BC partners had a negative association with time in relationship (rho = −0.33; p = 0.03) where cancer loneliness was higher when the relationship was shorter, but no associations with other baseline variables. Within couples, cancer loneliness was expressed by both partners in 62% of PC couples and 74% of BC couples.

**TABLE 2:** Cancer loneliness, mental, social and physical functioning scores in couples at baseline.

Characteristic	Prostate cancer couples				Breast cancer couples			
	Survivor		Partner		Survivor		Partner	
	(n = 75)		(n = 73)		(n = 44)		(n = 43)	
	Mean (SD) or %	Range	Mean (SD) or %	Range	Mean (SD) or %	Range	Mean (SD) or %	Range
Cancer loneliness	1.7 (0.7)	1.0–3.7	1.8 (0.7)	1.0–3.6	2.3 (0.8)	1.0–4.1	1.8 (0.7)	1.0–3.6
Cancer loneliness >1	77.3%	​	74.0%	​	95.5%	​	76.7%	​
Anxiety (PROMIS)	46.1 (0.2)	37.1–66.6	49.2 (7.8)	37.1–66.6	51.4 (8.7)	37.1–68.7	49.5 (9.6)	37.1–73.0
Depressive symptoms (CES-D)	7.2 (7.3)	0.0–30.0	7.1 (6.8)	0.0–34.0	10.8 (9.6)	0.0–40.0	9.3 (8.6)	0.0–37.0
Social function (SF-36)	51.9 (7.9)	24.1–56.9	52.1 (7.5)	24.1–56.9	47.1 (8.3)	24.1–56.9	50.3 (9.6)	24.1–56.9
Physical function (SF-36)	51.6 (5.5)	36.0–57.0	51.9 (5.7)	29.7–57.3	48.7 (8.0)	21.3–57.0	52.3 (10.3)	23.4–57.0
Bodily pain (SF-36)	50.2 (8.6)	33.0–62.1	50.5 (8.0)	33.0–62.1	49.0 (7.6)	29.2–62.1	51.9 (7.8)	37.2–62.12

**TABLE 3:** Correlations (Spearman’s rho) between cancer loneliness and continuous baseline characteristics.

Characteristic	Prostate cancer couples		Breast cancer couples	
	Survivor (n = 75)	Partner (n = 73)	Survivor (n = 44)	Partner (n = 43)
*Demographics and Health History*				
Age	−0.30**	−0.10	−0.12	−0.17
Time in relationship (years)	−0.07	−0.14	0.04	−0.33*
Comorbidities (charlson comorbidity Index)	0.11	−0.04	−0.14	−0.10
Body Mass Index (BMI; kg/m^2^)	0.09	−0.10	−0.01	0.06
Time since diagnosis (months)	0.05	—	0.31*	—
*Quality of Life*				
Anxiety	0.66**	0.32**	0.53**	0.36*
Depressive symptoms	0.61**	0.38**	0.55**	0.58**
Social function	−0.56**	−0.23	−0.56**	−0.34*
Physical function	−0.22	0.12	−0.41**	0.48
Bodily pain	−0.23*	0.05	−0.34*	−0.10

*p < 0.05.

**p < 0.01.

### Correlations between cancer loneliness and mental and physical health within each partner among PC and BC couples

Among PCS, cancer loneliness was significantly and positively associated with higher anxiety (rho = 0.66, p < 0.01), worse depressive symptoms (rho = 0.61, p < 0.01) and pain (rho = 0.23, p = 0.04) and inversely with social functioning (rho = −0.56, p < 0.01) (Table 3). Among BCS, cancer loneliness was significantly and positively associated with higher anxiety (rho = 0.53, p < 0.01), worse depressive symptoms (rho = 0.55, p < 0.01) and pain (rho = 0.34, p = 0.02) and inversely with social (rho = −0.56, p < 0.01) and physical (rho = −0.41, p = 0.01) functioning. Similar to their PCS spouses, cancer loneliness was positively associated with higher anxiety (rho = 0.32, p = 0.01) and worse depressive symptoms (rho = 0.38, p < 0.01) in PC partners, but in contrast not with pain or social or physical functioning. Similar to their BCS spouses, BC partners’ cancer loneliness was positively associated with higher anxiety (rho = 0.36, p = 0.02), worse depressive symptoms (rho = 0.58, p < 0.01) and inversely with social functioning (rho = −0.34, p = 0.03), but in contrast, not with pain nor physical functioning.

### Changes in cancer loneliness over 6 months of exercise in PCS and PC partners

**FIGURE 2:**
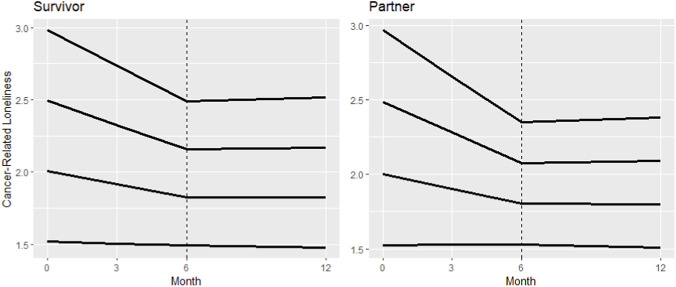
Change in cancer loneliness as a function of baseline cancer loneliness among prostate cancer survivors (left) and prostate cancer partners (right) participating in a six-month exercise program (all study arms combined). Each line represents predicted longitudinal trajectories at four different levels of baseline cancer loneliness based on data from PC couples.

**FIGURE 3:**
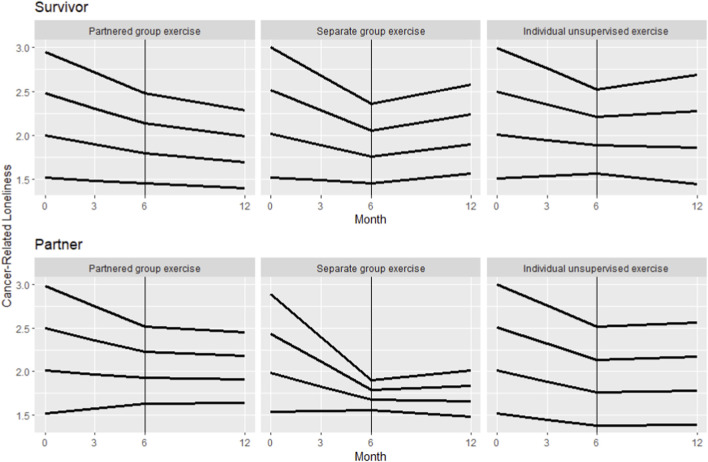
Change in cancer loneliness as a function of baseline cancer loneliness among prostate cancer survivors (top panel) and their partners (bottom panel) participating in one of three dyadic exercise programs (Exercising Together (partnered exercise) in a group, exercising separately in a group with other survivors or partners, or unsupervised independent exercise at home). Each line represents predicted longitudinal trajectories at four different levels of baseline cancer loneliness based on data from PC couples. Data are for visual comparison only as no inferential statistics were performed.

Among PC couples that provided longitudinal loneliness data, there was a significant interaction between the degree of change in cancer loneliness over the six-month exercise period and baseline loneliness for both PCS and PC partners with all exercise groups combined (Table 4). Declines in cancer loneliness were greater in PCS and PC partners with higher levels of loneliness at enrollment (both p < 0.01; Figure 2). For every 1-point increase in baseline cancer loneliness, there was an additional 0.31-point decrease (95%CI= (−0.49, −0.14)) for PCS and 0.42-point decrease (95%CI=(-0.61, −0.22)) for PC partners. Among those couples where both partners reported cancer loneliness, it declined in both partners in 29% of couples and declined in at least one partner in 55% of couples. The remaining 16% of couples whose cancer loneliness did not change had lower average baseline loneliness within the couple than those who responded more to the intervention. During the follow-up period, loneliness began to rise again but stayed below baseline values among couples reporting loneliness scores >1 at enrollment. Based on visual comparison of changes in cancer loneliness across the three exercise groups, cancer loneliness declined in all three groups in both PCS and PC partners. However, declines in loneliness appeared steepest among those participating in a supervised, group exercise class with other PCS or other PC partners, respectively, compared to exercising in a supervised group program together with other couples or exercising on their own at home (Figure 3). During the follow-up period loneliness appears to have leveled off within PC partners, whereas in PCS loneliness steadied or slightly rebounded in the individual unsupervised group and PCS only group but continued to slowly decline in PCS assigned to the Exercising Together arm.

**TABLE 4:** Longitudinal change in cancer loneliness (CL) over 6 months of exercise and a 6 months follow-up period as a function of baseline loneliness within PC couples.

Change over 6-month	Baseline cancer-related loneliness				
	1.0	1.5	2.0	2.5	3.0
	Estimate (95% CI)	Estimate (95% CI)	Estimate (95% CI)	Estimate (95% CI)	Estimate (95% CI)
Survivor					
0–6 months^a^	0.07 (−0.10, 0.23)	−0.09 (−0.21, 0.03)	−0.25 (−0.38, −0.11)	−0.40 (−0.59, −0.21)	−0.56 (−0.82, −0.29)
6–12 months	−0.03 (−0.19, 0.13)	−0.01 (−0.13, 0.10)	0.00 (−0.13, 0.13)	0.01 (−0.17, 0.19)	0.02 (−0.23, 0.28)
Spouse					
0–6 months^b^	0.28 (0.09, 0.47)	0.07 (−0.07, 0.21)	−0.13 (−0.28, 0.01)	−0.34 (−0.54, −0.14)	−0.55 (−0.82, −0.27)
6–12 months	−0.04 (−0.20, 0.13)	−0.02 (−0.14, 0.10)	0.00 (‘-0.13, 0.12)	0.01 (−0.16, 0.18)	0.03 (−0.21, 0.27)

^a^ Change in CL, for from 0–6 months changes significantly (p = 0.001) by −0.31 (−0.49, −0.14) for every 1-point increase in baseline CL.

^b^ Change in CL, for from 0–6 months changes significantly (p = 0.00004) by −0.42 (−0.61, −0.22) for every 1-point increase in baseline CL.

## Discussion

We found that among a subsample of married couples coping with PC or BC who were participating in our clinical exercise trial, 77%–96% of survivors and 74%–77% of care partners reported having at least some level of loneliness related to their own or their partners cancer. Loneliness was reported by both partners in 61% of PC couples and 74% of BC couples pointing to the interconnectedness of couples’ experience. While marriage is often a protective factor against general loneliness (Beutel et al., 2017), the toll of an illness may erode the support and emotional intimacy expected in intimate relationships. Which can exacerbate feelings of loneliness specific to the experience of having or caring for someone with cancer (Niedling and Hamel, 2023). In our parent study, we aimed to target couples who were within 3 years of one partner’s cancer diagnosis–a time period when the emotional cost of adjusting to cancer can overwhelm both partners, and lead to unique experiences of loneliness associated with a sudden relational shift into their roles as a patient or caregiver (Howden et al., 2022; Rollero, 2016). Since each partner’s loneliness may be unique to their new role in the context of one person’s chronic illness, they may become disconnected making it more difficult for them to be a source of effective social support for each other. In PC couples, we observed decreases in cancer loneliness over 6 months of exercise, particularly for persons experiencing more loneliness, suggesting that participating in a shared behavior together may have helped rebuild connections within the couple (Wilson et al., 2024). When visually comparing patterns across the different formats of social exercise or individual exercise, there appeared a possible benefit of specific social support when exercising with other survivors or care partners.

In our subsample of PCS and BCS participants, over three-quarters reported feeling some level of cancer loneliness. People with cancer are at higher risk of loneliness due to illness-related constraints on access to social networks, inadequate or unsatisfying social support, and existential threats (Miaskowski et al., 2021; Smith et al., 2024; Wheldon et al., 2025). In a 2023 report from the American Cancer Society on social isolation in cancer survivors within 7 years of their diagnosis (n = 1,555), 52% reported that they felt more isolated (including feeling lonely, left out, or lacking companionship) as a result of their cancer diagnosis, where persistent physical symptoms related to cancer had the most negative impact on social connection (American Cancer Society Cancer Action Network, 2023). Survivors in our study reported even higher rates, mirroring the rates we saw in another trial we conducted during a similar time frame (Winters-Stone et al., 2025). In our sample, loneliness was higher among PCS who reported being on androgen deprivation therapy (ADT), a common treatment for advanced PC that negatively impacts physical and mental health. PCS with poorer health and more advanced disease report greater feelings of loneliness (Helgason et al., 2001), which might be due to the isolation from social networks caused by persistent symptoms and side effects and worry about dying (Adams et al., 2017). Older age is typically associated with a greater risk of general loneliness due to shrinking social networks (Huxhold and Fiori, 2024/06) but in our sample, age was inversely associated with loneliness in PCS, possibly due to greater feelings of the existential threat of cancer when it strikes at a younger than typical age and a lack of specific social support networks for younger PCS (Chambers et al., 2015). Nearly all BCS in our study reported some level of cancer loneliness, which worsened with increasing time since diagnosis. A 2025 report using the HINTS survey data among 1,234 cancer survivors, reported that survivors who were >5 years past diagnosis were at 1.62-fold higher risk of having moderate-severe loneliness than survivors closer to diagnosis (Wheldon et al., 2025). As survivors transition out of cancer care they may have less cancer-specific social support and develop increasing fear of recurrence that worsen feelings of loneliness (Koch et al., 2014).

Rates and levels of cancer loneliness were higher for BCS than PCS. In studies of cancer survivors, women may trend toward reporting more loneliness than men (Smith et al., 2024). Differences in our study might be related to differences in how men and women experience loneliness, in how much they recognize and disclose feelings of loneliness, or because of differences related to their distinct cancers and/or the gender of their care partner. PCS report feeling stigma, shame and embarrassment due to changes in their perceived body image and physical function, which in turn can cause men to feel alone, unsupported and more likely to isolate themselves socially from friends and family (Eymech et al., 2022). PCS also express relational difficulties due to feelings of anger that further isolate them from support networks (Rice et al., 2021). Despite generally having larger social networks, BCS may also feel more isolated from friends and family due to their cancer experience, time demands of treatment, and physical and emotional symptoms (American Cancer Society Cancer Action Network, 2023; Shankar et al., 2011; Kobayashi and Steptoe, 2018). In a qualitative study, BCS referred themselves as having “survivor loneliness”, where they felt alone because they were more aware of their mortality, burdened by ongoing symptoms, and had an altered sense of identity and self (Rosedale, 2009). While nearly all BCS in our study reported some cancer loneliness a high proportion of PCS did too, suggesting that both men and women are subject to feeling socially isolated because of their cancer but also suggesting that intervention strategies should consider the potential unique drivers of cancer loneliness between genders.

Among couples in our study, three out of four partners reported loneliness related to their partner’s cancer. Caregivers often experience distress parallel to that of the patients, illustrating a reciprocal dynamic that may lead to interdependent distress (Jacobs et al., 2017). This is not surprising since cancer care partners can experience substantial loneliness due to the demanding nature of caregiving, social isolation, and the emotional toll of dealing with their partner’s illness (Gray et al., 2020). While care partners strive to provide support for their ill loved one, they may simultaneously lack social and emotional support themselves, magnifying feelings of loneliness which can impact their own wellbeing and the quality of care they can provide (Luszczynska et al., 2007; Peters et al., 2022). Loneliness from the caregiving experience may also transcend life stage as BC couples were still of working age compared retirement-aged PC couples. Among PC partners, loneliness was inversely associated with education. Persons with lower socioeconomic status have been shown to be at higher risk of loneliness, which may be due to systemically lower social support and less well-connected social networks, higher risk of poor health, or greater financial strain (Wang et al., 2024; Algren et al., 2020). BC partners reported higher loneliness when their partner was diagnosed earlier in their relationship. Care partners may feel more acute stress in response to an unexpected and sudden shift to a caregiving role that also separates them from their usual social networks (Luszczynska et al., 2007), while having few resources available to them within oncologic care. Since most BC partners were men, they may also lack the same degree of social networks to draw upon for support early in the disease trajectory and may be less inclined to feel the need to and/or seek support (Mahalik et al., 2007). Though attention is appropriately focused on the patient during the initial courses of cancer treatment, the healthcare system should recognize that care partners can also experience social isolation which may affect the relationship and sense of connection with their partner and ultimately, the quality of care they can provide.

Higher levels of cancer loneliness among survivors, regardless of cancer type, were associated with poorer emotional and social functioning and more pain interference. In people with cancer, social isolation and loneliness can adversely impact emotional wellbeing and are associated with higher levels of systemic inflammation and poorer immune functioning, fatigue, pain, sleep disturbance, anxiety and depressive symptoms, suicidal ideation (Emslie et al., 2009) and all-cause mortality (Jaremka et al., 2014; Jaremka et al., 2013; Du et al., 2021; Drageset et al., 2013). The relationship between loneliness and pain interference among survivors could suggest that uncontrolled pain contributes to social isolation because it may limit access to social networks, though it’s possible that loneliness may keep survivors from seeking out better symptom management leading to worse pain (Adams et al., 2018). These interdependencies may also explain the relationship between loneliness and poor physical functioning among BCS, who may be more socially limited because of poor mobility or who may engage in less physical activities if they feel socially isolated (Smith et al., 2024). Increasing loneliness among care partners was associated with worsening emotional wellbeing. Caregivers experience higher rates of depression and anxiety than non-caregivers, sometimes even more so than their ill partners (Litzelman et al., 2021; Litzelman et al., 2016). BC, but not PC partners also reported worsening social functioning with increasing loneliness, again pointing to the likelihood that men may have fewer social networks to begin with and may be less apt to access or seek social support because of the stigma over mental health and concern to not burden others with their socioemotional needs. Although the temporal relationships among mental health and loneliness in our study could not be clearly established, interventions aimed at emotional or social health might positively impact the other. Furthermore, appropriate and timely symptom management and physical rehabilitation for survivors may avoid a downward spiral that could lead to further social isolation and increasing dependence.

Among the 75 PC couples with longitudinal data 6 months of group exercise alleviated feelings of loneliness in both survivors and partners who reported cancer loneliness to begin with, and the benefits were greater in those with more loneliness. Exercise helped at least one and sometimes both partners feel less lonely in an overwhelming majority of couples. Furthermore, the effect continued after participation after the exercise program ended, suggesting some lasting benefit. People may have maintained their social ties with others in the program and/or may have reconnected with their partner, creating a sustained level of social support. There are a paucity of interventions aimed to reduce social isolation and loneliness in people with cancer, none that simultaneously address physical health and none that target the care partner too (McE et al., 2021). All couples in this sample had agreed to engage in a structured dyadic exercise program aimed to improve their physical, mental, and relational health. Thus, by virtue of participation, couples were engaged in some level of dyadic coping, where partners collaborate to manage the emotional and practical challenges posed by cancer (Lyons, 2020). This in and of itself may potentially mitigate some of their individual feelings of loneliness, as relationships characterized by communication and mutual support are associated with reduced feelings of isolation (Stefanut et al., 2021). An exercise program may have provided the couple with a shared experience where they could provide mutual support to one another, in turn making the couple feel more united in coping with the illness experience together (Kayser et al., 2007). Exercise can also reduce anxiety and depressive symptoms for both partners and improve their adjustment to cancer, which may reduce feelings of loneliness and may have helped partners feel less worried about each other’s health. Though sample sizes were too small to statistically test for differences in rates of change in loneliness across the different types of exercise programs, there seemed to be a slightly greater benefit for PC couples who participated in the study arm where survivors exercised with other survivors and partners with other partners. Social interactions outside of the marriage can significantly shape feelings of loneliness among spouses (Ermer et al., 2020). When couples have limited support systems or isolating experiences, partners may each begin to experience loneliness, creating a cycle of mutual loneliness that is challenging to break. Exercising with other survivors or care partners could have provided more role-specific and positive social support that helped alleviate the isolation that occurs as each partner copes with the chronic illness. We observed a similar benefit of supervised group exercise training in a different trial by our team, focused only PCS (Winters-Stone et al., 2025), demonstrating the reproducibility of our findings. PC partners also seemed to particularly benefit from exercising with other partners, possibly because there is a general lack of support offered to care partners within the healthcare system and women may have provided each other with peer-to-peer emotional support (Shankar et al., 2011). Due to the limitations in our current analysis, future studies are needed to further study the potential benefits of exercise.

This ancillary analysis on cancer loneliness among couples coping with prostate and breast cancer had strengths and limitations. Our findings are among the first reporting on cancer loneliness, a unique experience of loneliness related to cancer among both cancer survivors and their intimate partners. Our focus on the construct of cancer loneliness and the strong link to emotional and physical health may uncover a significant unmet need for survivors and care partners during the time couples are actively adjusting to and coping with cancer. We were also able to explore a potential benefit of exercise for couples experiencing cancer loneliness, an intervention that would also benefit their physical and mental health. Our small sample size within study arms can only point to ideas about the ideal way to deliver exercise to the couple but underscores the need for further study. Our study had limitations, including the relatively small sample and that we could not determine the direction of relationships between cancer loneliness and physical, emotional and social health. We had no control group that did not receive exercise thus it is possible that simply participating in the trial alleviated feelings of loneliness within couples. We also lacked data to confirm whether BC couples would have similarly benefited from engaging in the study exercise programs. However, the replication of our findings across two trials in PCS and the similar changes observed in PC partners suggest that shared exercise might be a viable strategy to mitigate cancer loneliness among couples coping with cancer (Winters-Stone et al., 2025).

To summarize, there needs to be greater recognition of loneliness within couples coping with cancer, particularly given that both loneliness and cancer disproportionately affect older adults but for whom relational isolation within a couple may be overlooked. Addressing loneliness in cancer care necessitates a dual focus on both the individual patient and the care partner as well as strategies that support rebuilding their connection to one another (Wilson et al., 2024). Couples engaging in a shared activity like exercise may help reconnect partners to each other–an effect that could be sustained over time. Based on findings from our studies, exercising with peers likely provides additional and unique social support that helps reduce feelings of loneliness related to the having or caring for someone with cancer. There is an urgent need to better understand and address this unmet need of loneliness within couples throughout the cancer experience. Exercise shows promising potential to address both the social, emotional, and physical needs of this at-risk population and warrants further study.

## Data Availability

The raw data supporting the conclusions of this article will be made available by the authors, without undue reservation.
